# Classification of fibroglandular tissue distribution in the breast based on radiotherapy planning CT

**DOI:** 10.1186/s12880-016-0107-2

**Published:** 2016-01-14

**Authors:** Prabhjot Juneja, Philip Evans, David Windridge, Emma Harris

**Affiliations:** North Sydney Cancer Center, Royal North Shore Hospital, Sydney, Australia; Institute of Medical Physics, School of Physics, University of Sydney, Sydney, Australia; Joint Department of Physics, The Institute of Cancer Research and The Royal Marsden NHS Foundation Trust, London, UK; Centre for Vision Speech and Signal Processing, Faculty of Engineering and Physical Sciences, University of Surrey, Guildford, UK

**Keywords:** Breast radiotherapy, Tissue segmentation, Fibroglandular tissue distribution

## Abstract

**Background:**

Accurate segmentation of breast tissues is required for a number of applications such as model based deformable registration in breast radiotherapy. The accuracy of breast tissue segmentation is affected by the spatial distribution (or pattern) of fibroglandular tissue (FT). The goal of this study was to develop and evaluate texture features, determined from planning computed tomography (CT) data, to classify the spatial distribution of FT in the breast.

**Methods:**

Planning CT data of 23 patients were evaluated in this study. Texture features were derived from the radial glandular fraction (RGF), which described the distribution of FT within three breast regions (posterior, middle, and anterior). Using visual assessment, experts grouped patients according to FT spatial distribution: sparse or non-sparse. Differences in the features between the two groups were investigated using the Wilcoxon rank test. Classification performance of the features was evaluated for a range of support vector machine (SVM) classifiers.

**Results:**

Experts found eight patients and 15 patients had sparse and non-sparse spatial distribution of FT, respectively. A large proportion of features (>9 of 13) from the individual breast regions had significant differences (*p* <0.05) between the sparse and non-sparse group. The features from middle region had most significant differences and gave the highest classification accuracy for all the SVM kernels investigated. Overall, the features from middle breast region achieved highest accuracy (91 %) with the linear SVM kernel.

**Conclusion:**

This study found that features based on radial glandular fraction provide a means for discriminating between fibroglandular tissue distributions and could achieve a classification accuracy of 91 %.

## Background

Radiotherapy is used to reduce the risk of local recurrence in early-stage breast cancer patients who have undergone breast-conserving surgery (BCS) [1]. The challenges of radiotherapy for early breast cancer are evolving from improving the basic survival rates to that of improving the quality of life of the survivors whilst maintaining local control. Partial Breast Irradiation (PBI) aims to irradiate only the volume of breast tissue surrounding the tumour bed (the region at higher risk of recurrence) rather than the whole breast to minimize radiation induced side effects [2]. The tumour bed target is defined during radiotherapy treatment planning using a computed tomography (CT) scans of the patient, usually in the supine position. A major challenge in PBI is the daily, or weekly, change in position, size and shape of the target region, which may lead to uncertainty in target localization prior to irradiation. To account for uncertainty in target localization a margin of normal tissue is included in the irradiated volume which increases the likelihood of side effects.

**Fig. 1:**
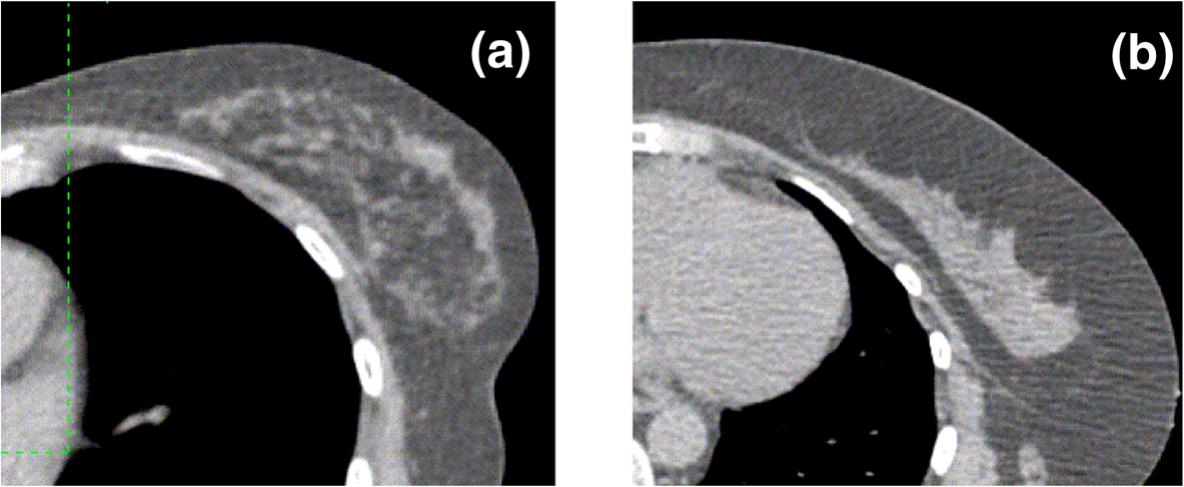
Sample fibroglandular distributions in the breast, middle-breast CT images: **a** Breast with sparse distribution of fibroglandular tissue, **b** Breast with non-spare distribution of fibroglandular tissue

This could be addressed using adaptive radiotherapy, based on biomechanical modelling of breast tissue [3]. Biomechanical modelling requires accurate segmentation of breast tissue into its constituent components: fibroglandular tissue (FT) and adipose tissue [3, 4]. Juneja et al. [3] demonstrated that the accuracy of breast tissue segmentation was affected by the spatial distribution of FT; accuracy was poorer in patients with sparsely distributed FT than in patients with non-sparsely distributed FT. It should be noted that the FT distribution is a physically different characteristic from the breast density or fibroglandular composition (FC). FC is the percentage of breast tissue that is fibroglandular, while FT distribution represents how the fibroglandular tissue is spatially distributed in the breast. Figure 1 illustrates different distributions (sparse and non-sparse) of FT in CT images acquired in two patients.

An automatic method is needed to assess FT distribution of the treated breast to identify breast cancer patients for whom the adaptive radiotherapy (ART) may be suitable. The radial glandular fraction (RGF) [5] is a convenient method to characterize the radial distribution of fibroglandular tissue. Previously, a study showed that RGF of the middle breast region was potentially useful for discrimination between fibroglandular tissue distributions [6]. The current study extends this work, using the same dataset, to evaluate RGF from other breast regions and to investigate RGF for classification of fibroglandular tissue distributions. The aim was to develop and evaluate a set of texture features, or metrics, derived from RGF, for their ability to classify the fibroglandular tissue (FT) distributions in the breast for breast ART. RGF was adapted for supine radiotherapy planning CT images. The ability of these features to classify FT distribution was tested against expert opinion. Classification performance was evaluated using the support vector machine with four different mapping kernels.

## Methods

### Patient dataset

The study datasets comprised planning CT scans of 23 patients. Datasets were originally collected for a comparison of prone and supine positioning for breast radiotherapy [7, 8] which was approved by the Royal Marsden Committee for Clinical Research and the National Health Service Regional Ethics Committee (London-Surrey Borders REC). Written informed consent was obtained from all the patients for participation in the study. Patients had undergone breast conservation surgery, during which time up to six pairs of surgical clips were placed to define the excision cavity boundaries. The patients received CT imaging for radiotherapy planning (from cervical vertebra 6 to below the diaphragm). FT distributions of the patients’ breast were visually assessed by an observer (EH) and grouped into non-sparse FT group (Group 1) and sparse FT group (Group 2). The grouping was reviewed and agreed by a consultant radiation oncologist specializing in breast radiotherapy [3].

### Radial Glandular function (RGF)

**Fig. 2:**
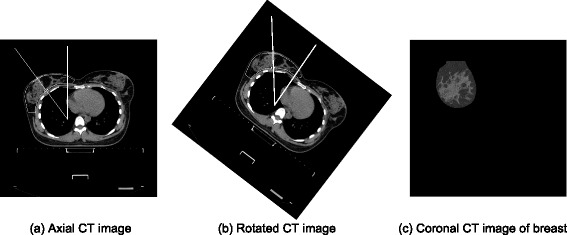
Pre-processing of supine breast data: **a** Original scan with vector showing orientation of center of breast; **b** Rotated image with center of breast at 0° to the vertical direction; **c** Coronal slice through **b**

The radial glandular fraction (*RGF*) presented by Huang et al. [5], was developed using coronal images acquired using breast CT with patients positioned prone. The patient datasets used in this study were axial CT images, acquired with patients positioned supine, which is standard practice for all the patients undergoing breast radiotherapy treatment. These data were processed to produce a breast orientation equivalent to that used by Huang et al. [5] using the following steps: (1) segmentation of the whole breast from the axial CT images using clinician outlining, and (2) transformation, resampling and rotation, using bilinear interpolation were applied to the segmented breast to obtain the desired image orientation. Prior to rotation the whole breast 3D data were re-sampled, to produce cubic voxels (1x1x1 mm^3^). The re-sampled breast was rotated about the superior-inferior axis by the acute angle formed between anterior-posterior axis and a line passing through the nipple and perpendicular to chest-wall, see Fig. 2.

**Fig. 3:**
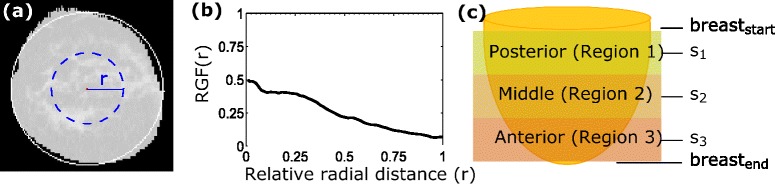
Description of radial glandular fractions (RGF) measurement: **a** Coronal CT image (processed, see Fig. 2): white circle encompasses the whole breast, and blue dotted circle is an example circle with relative radius (*r*) for which RGF(*r*) is calculated; **b** RGF of the slice in **a**; **c**. Breast is evenly divided into three regions: posterior (region 1), middle (regions 2), and anterior (region 3) middle slices s_1_, s_2_, and s_3_, respectively

For an image i, the breast radius, R, was calculated by equating the total area of breast tissue to the area of a circle (Fig. 3a). *RGF*^*i*^(*r*) of image i, was the fraction of pixels marked as FT on a circle with relative radial distance, *r*, and its center at the image center of mass (Fig. 3b). The relative radial distance, *r*, is the circle radius divided by the breast radius R. For each image, one hundred values of *r* were considered. The whole breast was evenly divided into three regions (Fig. 3c): the posterior breast (region 1), the middle breast (region 2), and the anterior breast (region 3). The RGFs of the three breast regions were calculated by averaging the *RGF*^*i*^(*r*) over five images centered on slice s (s_1_, s_2_, or s_3_). A fibroglandular tissue segmentation method recommended by a previous study [3] was utilized in the current study.

### RGF features

**Fig. 4:**
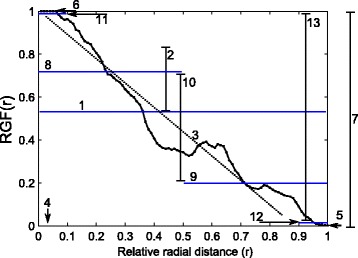
Example of radial glandular fraction (RGF) features (as listed in Table 1) that were evaluated for the classification of the fibroglandular distribution: (1) mean value and (2) standard deviation of the 100 RGF values, (3) slope of the linear fit of RGF values versus relative radius, (4) radial position (r) of the maximum RGF value, (5) the minimum value of RGF, (6) the maximum value of RGF, (7) the difference in the maximum and minimum values of RGF, (8) mean of RGF values for which relative radial distance was less than or equal to 0.5 (mean of inner 50 %), (9) mean of RGF values for which relative radial distance was greater than 0.5 (mean of outer 50 %), (10) the difference in the mean of inner and outer 50 %, (11) mean of the highest 10 % of RGF values (mean of highest 10 %), (12) mean of the lowest 10 % of RGF values (mean of lowest 10 %), and (13) the difference in the mean of highest and lowest 10 %

RGF gives the proportion of fibroglandular tissue in the breast as a function of relative radius, it may be considered a graphical representation of the FT distribution. For classification of the spatial distribution of fibroglandular tissue, metrics describing this distribution are needed. The RGF was characterized using the features listed in Table 1 and presented in Fig. 4. These features, explained below, were investigated in order to classify breast fibroglandular tissue distribution of individual patients. These features were: mean value and standard deviation of the 100 RGF values for the corresponding relative radial distances, slope of the linear fit of RGF values versus relative radius (r), radial position (r) of the maximum RGF value, the minimum value of RGF, the maximum value of RGF, the difference in the maximum and minimum values of RGF, mean of RGF values for which relative radial distance was less than or equal to 0.5 (mean of inner 50 %), mean of RGF values for which relative radial distance was greater than 0.5 (mean of outer 50 %), the difference in the mean of inner and outer 50 %, mean of the highest 10 % of RGF values (mean of highest 10 %), mean of the lowest 10 % of RGF values (mean of lowest 10 %), and the difference in the mean of highest and lowest 10 %.

**Table 1:** Radial glandular fraction (RGF) features evaluated for the classification of the distribution of fibroglandular tissue

Texture feature^a^	Feature number
Mean RGF	1
Standard deviation of RGF	2
Slope of linear regression of RGF vs. *r*	3
Radial position of maximum RGF	4
Minimum RGF	5
Maximum RGF	6
Difference in maximum and minimum	7
Mean of radially inner 50 % RGF	8
Mean of radially outer 50 % RGF	9
Difference in means of radially inner and outer 50 % RGF	10
Mean of highest 10 % RGF	11
Mean of lowest 10 % RGF	12
Difference in means of maximum and minimum 10 % RGF	13

^a^Features were calculated using RGF values (100) and the corresponding relative radial distances (*r*)

### SVM classifier

The support vector machine (SVM) [9, 10], a widely-used classifier, was used to evaluate the classification performance of the RGF features. The SVM constructs a maximum-margin hyper-plane in the high dimensional input feature space, linearly separating the data points into two classes ensuring the maximum gap between the classes. Though linear, this decision-boundary can be rendered arbitrarily-convoluted with respect to the input space via the *kernel-trick*, in which inner-product relations within the SVM optimization function are replaced by kernel functions, replicating the effect of a *feature mapping*. In this study, four kernels were chosen for evaluation within the SVM to cover a representative range of behaviors; polynomial, radial basis function and sigmoid kernels map the features into Hilbert spaces with differing characteristics, while the linear kernel equates to retaining the existing feature space and the radial basis function kernel maps into an infinite dimensional Hilbert space thereby guaranteeing linear class separability on the training data. The sigmoid kernel derives historically from work on Neural Networks, and exhibits an inherent quasi-classification-like aspect that differentiates it from the other kernels. It should be noted that it is not possible to say a priori which kernel will be better.

Classification performance for a group of features was measured with leave-one-out cross-validation for all four mapping kernels: linear, polynomial of order 3, radial basis function (RBF) with sigma value of 1 and sigmoid. The overall *C* parameter for the SVM was 1. Leave-one-out cross-validation used a single observation from the original data as the validation set, and the remaining observations as the training set. This was repeated such that each observation in the original data was used once as the validation set.

### Analysis

For each patient dataset, the texture features, listed in Table 1, were calculated and evaluated for their ability to classify the FT spatial distribution. RGF features of the three breast regions for these patient groups were evaluated. The differences in the fibroglandular composition (FC) (percentage of FT) of the breast in the two groups were compared using the Wilcoxon rank sum test. For each feature, the two group means were calculated and compared using the Wilcoxon rank sum test to investigate their discriminative power. Four SVM classifiers with different mapping kernels were used to evaluate the classification performance of the 13 RGF features together. Classification performance was evaluated for the features from each of the three individual breast regions and all the regions combined together. Performance accuracy was calculated as a percentage of true identifications (both sparse and non-sparse) out of total identifications.

## Results

Expert ranking found 15 patients with non-sparse FT distribution and eight with sparse FT distribution. There was no statistically significant difference in the FC (percentage of fibroglandular tissue) of breasts between the two groups (*p* = 0.50).

### Radial glandular fraction (RGF)

**Fig. 5:**
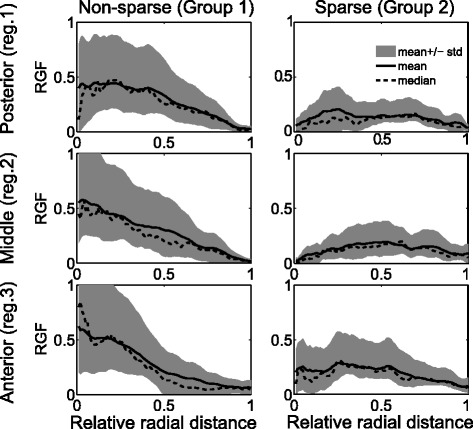
Mean radial glandular fraction (averaged across all patients in a group) (RGF) are plotted for the three breast regions: (posterior: 1, middle: 2, and anterior: 3) for the two groups: non-sparse and sparse

The group means (averaged over all patients in a group) RGF for the three breast regions for the two groups (non-sparse and sparse) are presented in Fig. 5. The variation of RGF with radius differed qualitatively between the two groups in all three breast regions (Fig. 5). For group 1 (non-sparse group) mean RGF varied with relative radius, such that RGF was highest at the center (*r* = 0) and lowest at the periphery (*r* = 1) of the breast. While for the sparse breast, the variability in the RGF with relative radius was small. In both the groups, the anterior region had the highest average RGF value near the center of the breast (*r* = 0).

### RGF feature analysis

**Fig. 6:**
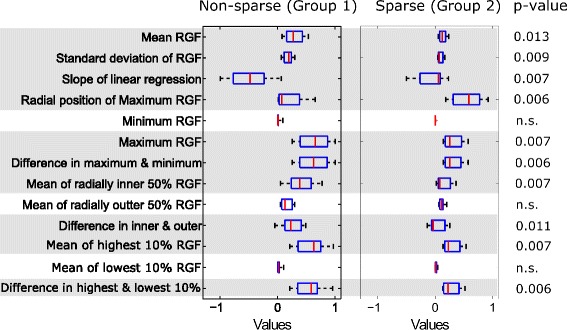
Texture features from the posterior region. Box plots of texture features from the posterior region for two groups: non-sparse and sparse. In each box, median is marked by a central mark (*red*), the 25^th^ and 75^th^ percentile are the edges of the box (*blue*), and error bars represent the *range*. Features which had significant differences (*p* <0.05) are highlighted in *grey*

**Fig. 7:**
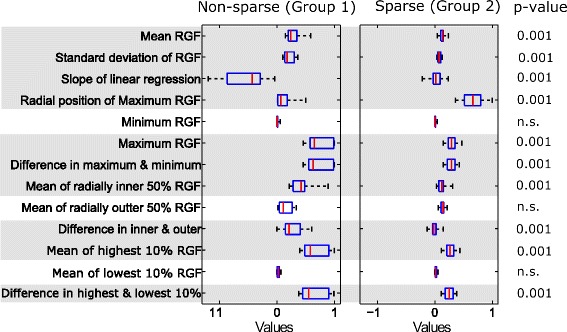
Texture features from the middle region. Box plots of texture features from the middle region for two groups: non-sparse and sparse. In each box, median is marked by a central mark (*red*), the 25^th^ and 75^th^ percentile are the edges of the box (*blue*), and error bars represent the range. Features which had significant differences (*p* <0.05) are highlighted in *grey*

**Fig. 8:**
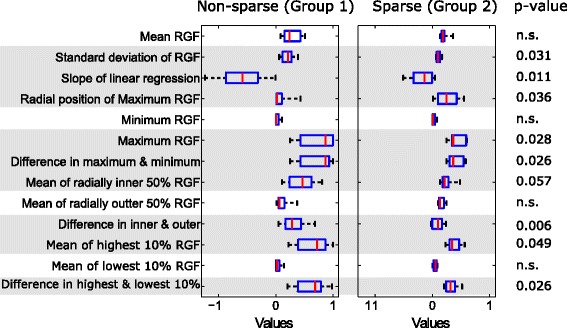
Texture features from the anterior region. Box plots of texture features from the anterior region for two groups: non-sparse and sparse. In each box, median is marked by a central mark (*red*), the 25^th^ and 75^th^ percentile are the edges of the box (*blue*), and error bars represent the range. Features which had significant differences (*p* <0.05) are highlighted in *grey*

The differences in the texture features between the two groups for posterior, middle and anterior breast regions are given in the box plots in Figs. 6, 7 and 8, respectively. The middle region had the largest proportion (10 of 13) of features with the most significant differences (*p* <0.001) between groups. The anterior and posterior region had 9 and 10 features, respectively, with statistically significant differences (*p* <0.05). RGF features from the middle regions had the highest discriminative power followed by posterior and anterior regions.

The features with significant difference (*p* <0.05) between groups were: mean value (mean of RGF) and standard deviation of the 100 RGF values for the corresponding relative radial distances, slope of the linear fit of RGF values versus relative radius (r), radial position (r) of the maximum RGF value, the maximum value of RGF, the difference in the maximum and minimum values of RGF, mean of RGF values for which relative radial distance was less than or equal to 0.5 (mean of inner 50 %), the difference in the mean of inner and outer 50 %, mean of the highest 10 % of RGF values (mean of highest 10 %), and the difference in the mean of highest and lowest 10 %.

### SVM classifier

**Fig. 9:**
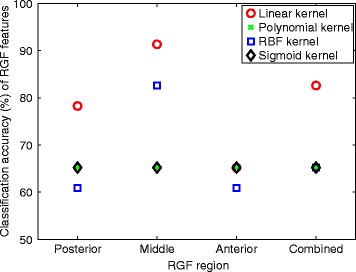
Classification performance results. Classification performance of radial glandular fraction (RGF) features for different breast regions, posterior, middle, anterior, and all regions combined, for linear, polynomial, radial basis function (RBF), and sigmoid SVM kernels

RGF feature classification performance for individual breast regions and all the regions combined and for linear, polynomial, radial basis function (RBF), and sigmoid SVM kernels are given in Fig. 9. For all four SVM kernels, the middle breast region gave the highest classification accuracy (percentage of true identifications). It should be noted that the middle region was found to have the highest discriminative power, as shown in Figs. 6, 7 and 8. For all regions, the linear kernel gave the highest accuracy and gave 91.3 % in the middle region. The polynomial and sigmoid kernels gave 66 % accuracy for all the breast regions. The accuracy of linear and RBF kernels varied with breast regions, from 62 to 91 %.

## Discussion

In this study, various texture features were evaluated to assess the spatial distribution of fibroglandular tissue (FT) in the breast. Results showed that the features derived from the radial glandular fraction (RGF) provide a means for discriminating between non-sparse and sparse groups. The study of the classification performance of these features using support vector machine (SVM) classifier gave promising results with accuracy as high as 91 %.

RGF features from the middle regions had the highest discriminative power. This is most likely due to the breast tissue architecture in the three regions. The anterior region had the lowest discriminative power between the groups. This may be because it is close to the nipple and a greater portion of fibroglandular tissue is located in the center of the anterior region of the breast in all cases [5]. Discriminative power within the posterior region may be decreased due to the smaller proportion of fibroglandular tissue compared to the middle region.

Sparseness of the FT distribution is of interest for the purpose of segmenting breast tissues and developing models for use in adaptive breast radiotherapy, as discussed in the introduction. Moreover, the availability of a means of quantifying the FT distribution can facilitate further studies. For example, studies of the association of the FT distribution with secondary breast cancer risk and radio-toxicity risk. To the best of our knowledge, no other study has ever studied texture features to classify the sparseness of FT based on CT data.

Tissue distribution patterns have been widely investigated on mammograms [11–14]. Li et al. [12] studied power spectrum analysis features on mammograms to differentiate between high-risk BRCA1/BRAC2 mutation carriers and low-risk women, and found statistically significant differences (*p* <0.0001). Manduca et al. [14] evaluated the association of various breast tissue texture features with the risk of breast cancer using mammograms of 768 women. They found features which predicted breast cancer risk at a similar magnitude as mammographic percentage density. Nie et al. [15], using breast magnetic resonance imaging (MRI), investigated features such as circularity, convexity, irregularity, and compactness to characterize morphology of FT distribution into intermingled (*sparse*) and central patterns (*non-sparse*).

A large proportion of features (9 to 10 out of 13) based on RGF from the individual breast regions had significantly different (*p* <0.05) values for the non-sparse and sparse groups. It should be noted that, the results for the middle region are the same as previously reported [6]. The RGF features were further investigated, for their classification performance using the support vector machine. The classification performance of the RGF features set was evaluated for three individual spatial regions and all the regions combined. It was found that the features from the middle breast provide most accurate classification of FT distributions. However a study needs to be performed to identify the best of combination of features for the task and improve classification accuracy.

This study investigated four commonly used SVM kernels for classification to cover a representative range of behavior. Each has advantages and disadvantages entirely specific to the classification problem and it is not possible to determine a priori which kernel would be most applicable in advance. In our evaluation, the fact that the linear SVM performs best of the tested kernels suggests that the input feature space is already sufficiently rich with good linear class separation without requiring mapping into an alternative Hilbert space.

Furthermore, classification accuracy was measured using leave-one-out cross-validation within the same dataset; to better evaluate the performance an independent test data is required. The data used in the study had a small number of cases, consisting of 15 non-sparse and eight sparse FT distributions. Also, the ground truth was based was based on one expert’s opinion. To quantify and minimize observer bias more than one expert would be required and possibly repeat ranking sessions. Because image quality can vary between datasets, the influence of image quality on FT classification should also be investigated.

## Conclusion

This study evaluated texture features derived from, the recently developed, RGF for classification of the spatial distribution of FT. Texture features, based on the radial glandular fraction are suitable for the classification of FT and gave accurate classification. Features derived from the middle breast region had the highest differentiating power.

## Abbreviations

FT: fibroglandular tissue
CT: computed tomography
RGF: radial glandular fraction
SVM: support vector machine
BCS: breast-conserving surgery
PBI: partial breast irradiation
FC: fibroglandular composition
ART: adaptive radiotherapy
RBF: radial basis function
MRI: magnetic resonance imaging
